# Genome Survey Sequencing of *Indigofera pseudotinctoria* and Identification of Its SSR Markers

**DOI:** 10.3390/genes16090991

**Published:** 2025-08-23

**Authors:** Jing Chen, Qifan Ran, Yuandong Xu, Junming Zhao, Xiao Ma, Wei He, Yan Fan

**Affiliations:** 1 Chongqing Academy of Animal Sciences, Rongchang, Chongqing 402460, China; chenjing9101@163.com (J.C.); ranqifan@outlook.com (Q.R.); 55813961@163.com (Y.X.); cqhewei1978@163.com (W.H.); 2 Chongqing Crop Germplasm Rongchang Forage Resource Bank, Rongchang, Chongqing 402460, China; 3 College of Grassland Science and Technology, Sichuan Agricultural University, Chengdu 611130, China; junmingzhao163@163.com (J.Z.); maxiao@sicau.edu.cn (X.M.)

**Keywords:** *Indigofera pseudotinctoria*, genome survey, next-generation sequencing, flow cytometry, microsatellite, gene functional annotation

## Abstract

Background: *Indigofera pseudotinctoria*, a traditional Chinese forage and medicine widely used in East Asia, holds significant economic and agricultural value. Despite this, genomic information regarding *I. pseudotinctoria* remains conspicuously lacking. Methods: In this study, we utilized genome survey sequencing to elucidate the complete genome sequence of this species. Results: The genome size of *I. pseudotinctoria* to be around 637–920 Mb with a heterozygosity rate of 0.98% and a repeat rate of 66.3%. A total of 240,659 simple sequence repeat (SSR) markers were predicted in the genome of *I. pseudotinctoria*. Substantial differences were observed among nucleotide repeat types, for instance, mononucleotide repeats were found to be predominant (62.47%), whereas pentanucleotide repeats were notably scarce (0.24%). Furthermore, among dinucleotide and trinucleotide repeats, sequence motifs AT/AT (66.57%) and AAT/ATT (54.15%) were found to be particularly abundant. Among the identified unigenes, 58,790 exhibited alignment with known genes in established databases, including 33,218 genes within the Gene Ontology (GO) database and 10,893 genes in the Kyoto Encyclopedia of Genes and Genomes (KEGG) database. Conclusions: This study marks the first attempt to both sequence and delineate the genomic landscape of *I. pseudotinctoria*. Importantly, it will serve as a foundational reference for subsequent comprehensive genome-wide deep sequencing and the development of SSR molecular markers within the scope of *I. pseudotinctoria* research.

## 1. Introduction

*Indigofera pseudotinctoria* (2n = 2x = 16), commonly known as “Chinese Indigo”, is a flowering plant species belonging to the genus *Indigofera* in the Fabaceae family (Leguminosae) [1]. Its native habitat spans East Asia, including specific regions in China, Japan, and Korea [2]. Historically, *I. pseudotinctoria* has been used as a natural dye source and as a bioaccumulate of materials such as lead and caumium [3,4]. Furthermore, *I. pseudotinctoria* holds additional importance as an agronomically and economically valuable perennial leguminous shrub. It is particularly renowned for its medicinal properties, which are attributed to its rich store of natural antioxidants, including flavonoids, polyphenol, and amino acids [5,6]. With these attributes, *I. pseudotinctoria* is a crucial asset within agricultural systems.

In recent years, driven by the increasing demand for natural medicinal plants, the scrutiny of *I. pseudotinctoria* has gained significant importance [7]. However, ongoing habitat degradation driven by human activities hampers efforts to meet increasing demand while maintaining sustainability [8]. As a result, the need to domesticate and cultivate this species has become a viable solution. With advances in cultivation techniques, the focus of future efforts is likely to shift towards the development of diverse varieties. Currently, the lack of genomic information hinders the full exploration and utilization of *I. pseudotinctoria*.

Due to their numerous advantages, such as robust reproducibility, co-dominance, substantial abundance, and straightforward applicability, SSR markers have become indispensable tools for analyzing of genetic diversity and conducting linkage mapping [9,10]. Both genomic SSRs and EST SSRs act as complementary resources in the field of plant genome mapping. Notably, recent efforts have included the meticulous selection and validation of 44 pairs of EST-SSR markers through transcriptome sequencing in *I. szechuensis* [11]. This project aimed to unravel the population’s genetic structure within *I. pseudotinctoria*. While EST-SSRs are useful for genetic analysis, they are somewhat limited by their relatively lower polymorphism and the higher likelihood of being scarce in non-coding genomic regions [12]. In contrast, genomic SSRs display greater polymorphism and a tendency for broader genomic distribution, which contributed to improved map coverage [13]. Regrettably, no markers based on genomic sequences are currently available for *I. pseudotinctoria*.

Next-generation sequencing (NGS) has emerged as a powerful and innovative approach for the efficient discovery of numerous simple sequence repeat (SSR) markers [9,14]. Compared to traditional methods, this technique not only dramatically enhances sequencing throughput but also significantly reduces both time consumption and overall experimental costs. By integrating NGS with K-mer frequency analysis, genomic survey analysis allows for the estimation of key genomic features, including genome size, GC content, heterozygosity level, and repeat sequence proportion [15,16]. This method has proven effective in accurately predicting whole-genome sizes across multiple plant species, including potato (*Solanum tuberosum*) [17], wheat (*Triticum aestivum*) [18], and rice (*Oryza sativa*) [19]. In the study, we applied genomic survey analysis together with flow cytometry to explore the genome architecture of *I. pseudotinctoria*. Our research was guided by three main objectives: firstly, to determine the genome size, GC content, and heterozygosity levels of *I. pseudotinctoria*; secondly, to analyze the distribution patterns of SSR motifs throughout its genome using survey sequencing data; and thirdly, to conduct functional annotation through Gene Ontology (GO), Kyoto Encyclopedia of Genes and Genomes (KEGG), and KOG pathways. The results obtained from this investigation contribute valuable insights into the genomic characteristics of *I. pseudotinctoria*, laying a solid foundation for future large-scale genome sequencing and genetic resource development.

## 2. Materials and Methods

### 2.1. Plant Materials

**Figure 1:**
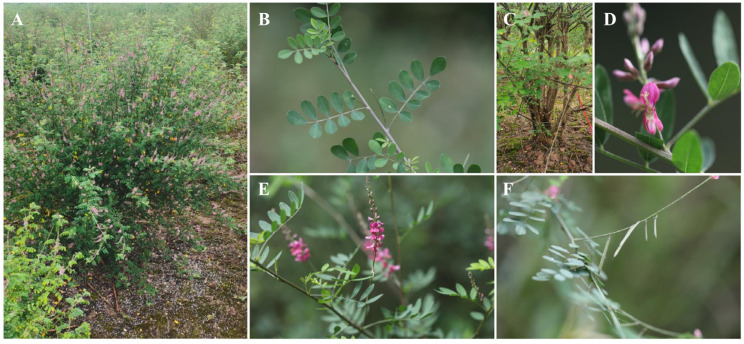
The morphological characteristics of *I. pseudotinctoria*. (**A**) The plant; (**B**) the leaves; (**C**) the tree trunk; (**D**) the flower; (**E**) the branch with inflorescence; (**F**) the branch with silique.

*I. pseudotinctoria* wild accessions were collected from Chongqing, China (107.37° N, 29.10° E). A selected individual plant (Germplasm ID: XKY20150201) was developed through multiple cycles of recurrent mass selection using progenitor germplasm derived from these wild accessions (Figure 1). The germplasm served as material for subsequent analyses, including flow cytometry, genome survey sequencing, and SSR characterization. Genomic DNA was extracted from youthful leaf tissues of *I. pseudotinctoria*, using a Tiangen Biotech (Beijing, China) plant genomic DNA extraction kit with the manufacturer’s stipulations. Subsequently, the integrity and concentration of the extracted DNA samples were evaluated via 1% agarose gel electrophoresis.

### 2.2. Genome Size Estimation by Fow Cytometry

The genome size was determined using the Beckman CytoFLEX^TM^ flow cytometer (Beckman, Pasadena, CA, USA). The protocol was appropriately optimized and modified based on the methods of Doležel and Bartoš [20]. The genome size estimation utilized rice (*O*. *sativa* subsp. *japonica* cv. Nipponbare) (2n = 2x = 24, 1C DNA content = 389 Mb, GC content = 43.6%) [21] as an internal reference standard. Approximately 100 mg of young leaves (3-week-old) was collected and immediately wrapped in moist filter paper, then stored in an icebox for subsequent use. For rice, 1 mL of pre-chilled OTTO’s I buffer was added. The tissue was quickly and finely chopped using a sharp single-edge razor blade, followed by incubation at 4 °C for 5 min. Then, 0.5 mL of OTTO’s II buffer was added to facilitate better nuclear release. For *I. pseudotinctoria*, 1 mL of LB01 buffer was added. The tissue was finely chopped using a sharp single-edge razor blade, followed by incubation at 4 °C for 5 min. Then, 0.5 mL of LB01 buffer was added. The lysate was filtered through a 300-mesh nylon mesh, and the filtrate was collected in a 1.5 mL centrifuge tube. The sample was centrifuged at 4 °C for 5 min at 1000 rpm/min. Then, 100 μL of the cell suspension was transferred to a sterile EP tube, mixed with 50 μL of propidium iodid (PI) staining solution, and incubated at 4 °C in the dark for 30 min. After staining, 1 mL of PBS was added to resuspend the cells, followed by centrifugation at 4 °C for 5 min at 1000 rpm/min. The supernatant was discarded, and the pellet was resuspended in 400 μL of PBS. The cell suspension was immediately subjected to flow cytometry analysis. Nuclear suspensions were prepared at a 1:1 (test:control) ratio for flow cytometric assays. A 488 nm green argon laser was employed to examine a minimum of 5000 nuclei per sample. Fluorescence detection and subsequent data processing were performed using Kaluza software version 3.1 (www.mybeckman.cn/flow-cytometry/software/kaluza, accessed on 30 March 2024), maintaining coefficient of variation (CV) values below 5% for both peaks. The nuclear DNA content of test samples was determined using the following computational approach: Estimated genome size = [(sample G0/G1 peak mean)/(standard G0/G1 peak mean)] × standard genome size.

### 2.3. Genome Survey Sequencing and Quality Control

A paired-end library with an insert size of 220 base pairs (bp) was constructed assembled through the controlled fragmentation of genomic DNA, meticulously following the standardized procedure established by Illumina (Beijing, China). Subsequent to the library’s preparation, the Nanjing Genepioneer Biotechnologies Co., Ltd. (Nanjing, China) employed an Illumina HiSeq 2500 sequencing platform to generate the sequence data. The process of Base Calling was executed utilizing Illunima Casava 1.8. After sequencing, filtering and sequence data correction yielded clean reads.

### 2.4. Genome Sequencing Assembly and GC Content

For the assembly of the genome, we employed SOAPdenovo2 software version 1.0 (https://github.com/aquaskyline/SOAPdenovo2, accessed on 20 February 2025) and AByss software version 2.3.10 (https://github.com/bcgsc/abyss, accessed on 20 February 2025) [22,23], utilizing clean reads. The assessment of K-mer sizes at 17 was executed with default parameters, and the optimal K-mer size was determined based on the N50 length [24]. Given that sequences under 200 bp were prone to originating from repetitive or low-quality sources, reads exceeding a length of 200 bases were selected for subsequent contig sequence realignment. Ensuring the concurrence of the paired-end relationship between reads and contigs, scaffolds were progressively constructed utilizing paired-end inserts. To yield insights into the genome’s characteristics, we computed the average depth and GC content for each window, producing both GC-depth plots and determining repeat content by stratifying GC clusters [25]. BUSCO software version 6.0 was employed to quantitative assessment of genome assembly and annotation [26].

### 2.5. Genomic SSR Identification and PCR Amplification

The identification of SSR markers was carried out using MISA software (version 2.1, http://pgrc.ipk-gatersleben.de/misa/, accessed on 15 March 2025). SSR motifs identification was based on the following thresholds: The minimum repeat number for mononucleotide motifs was set at **ten**, for dinucleotide motifs at **six**, and for trinucleotide, tetranucleotide, pentanucleotide, or hexanucleotide motifs, the threshold was **five**. The compound SSRs were identified as sequences where distinct microsatellite repeats.

In this study, due to the abundance of SSRs, 17 SSR loci were randomly selected from tri- and tetranucleotide microsatellites and designed corresponding primer pairs for PCR amplification. These primer pairs were designed using Primer Premier 5.0 software (www.premierbiosoft.com/primerdesign/, accessed on 30 May 2025) (Table 1), with all oligonucleotides conforming to the following specifications: length between 20 and 26 nucleotides, amplification product size ranging from 100 to 300 base pairs, and optimal annealing temperatures maintained at 50–60 °C. Primers were synthesized by Zhejiang Youkang Biotechnology Co., Ltd., Huzhou, China. The 20 µL PCR reaction system contained 1 µL genomic DNA (40 ng/µL), 0.5 µL (20 pmol/L) of each forward and reverse primer, 10 µL of 2X Taq-AS PCR Mix (BestEnzymes Biotech, Lianyungang, China), and 8 µL of double-distilled water. The PCR conditions were as follows: 2 min at 94 °C; 30 cycles at 94 °C for 20 s, 60 °C for 20 s, and 72 °C for 20 s; and a final extension at 72 °C for 3 min. The final PCR product was visualized by 2% agarose gel electrophoresis at 120 V for 30 min.

### 2.6. Gene Prediction and Annotation

We accessed the high-quality unigene library through using Trinity software v2.15.1 [27]. Following its acquisition, the obtained unigene underwent comprehensive bioinformatics analysis, encompassing functional annotation and categorization. To facilitate this, we employed the BLASTx comparison tool (https://blast.ncbi.nlm.nih.gov/Blast.cgi?PROGRAM=blastx&PAGE_TYPE=BlastSearch&LINK_LOC=blasthome, accessed on 20 April 2025) for unigene-protein database comparison (with an E-value threshold of ≤ 1 × 10^−5^) [28]. The functional annotation was contingent upon the resemblance of the gene to the functional annotation information for the encoded protein of the unigene. The protein databases employed for this purpose comprised NR (Non-Redundant Protein Database) (https://www.ncbi.nlm.nih.gov/protein, accessed on 20 April 2025), KOG (Clusters of Orthologous Groups for Eukaryotic Complete Genomes) [29], GO (Gene Ontology Database) [30,31], and KEGG (Tokyo Encyclopedia of Genes and Genomes) [32].

## 3. Results

### 3.1. Genome Size Estimation by Fow Cytometry

**Figure 2:**
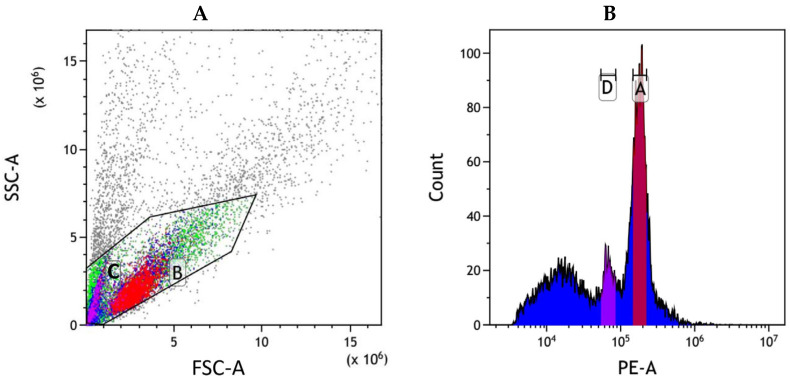
Genome size of *I. pseudotinctoria* analyzed by flow cytometry, with rice as the internal control. (**A**) Flow scatter diagram of cell mixed suspension. The *x*-axis represents the signal of forward scatter area (FSC-A), the *y*-axis represents the signal of side scatter area (SSC-A). Region B (red): G0/G1 nuclei of *I. pseudotinctoria*. Region C (pink purple): G0/G1 nuclei of rice. (**B**) Histogram of relative fluorescence intensity derived from nuclei isolated from rice and *I. pseudotinctoria* processed simultaneously. The *x*-axis represents the fluorescence intensity of PI fluorescence area (PE-A), the *y*-axis represents the number of cells. Peak A (rose red): G0/G1 nuclei of *I. pseudotinctoria*, Peak D (purple): G0/G1 nuclei of rice.

The application of flow cytometric analysis produced an intricate histogram with high resolution (Figure 2). Both rice and *I. pseudotinctoria* exhibited sharp and narrow peaks without overlapping interference between their measurement positions. The distinct particle clusters demonstrated excellent separation between the two species, confirming the reliability of using rice as an internal reference. The mean coefficient of variation (CV) of *I. pseudotinctoria* and the internal standard rice were quantified at 4.73% and 3.56%, respectively (Table 2). The outcomes unveiled that the genome size of *I. pseudotinctoria* approximated 920 ± 2 Mb.

### 3.2. Genome Sequencing and Sequence Assembly

A total of 48.952 Gb of clean bases were successfully acquired, with Q20 and Q30 values of 98.18% and 93.51%, respectively (Table 3). The cumulative count of sequences amounted to 553,021, encompassing a total length of 431,452,197 base pairs (bp) (Table 4). Within the genome of *I. pseudotinctoria*, the most extensive assembled sequence extended over 90,236 bp, while the N50 length reached 3506 bp. All clean reads was employed as query sequences for BLAST (Basic Local Alignment Search Tool) analysis against the NCBI’s (National Center for Biotechnology Information) Nucleotide Sequence Database (NT). The outcome of this analysis pinpointed the top five comparative species: *Abrus precatorius* (9393; 15.98%), *Camellia sinensis* (8869; 15.09%), *Spatholobus suberectus* (5615; 9.6%), *Glycine soja* (4172; 7.10%), and *Trifolium pratense* (3355; 5.70%) (Figure S1).

### 3.3. Genome Size Estimation and GC Content

**Figure 3:**
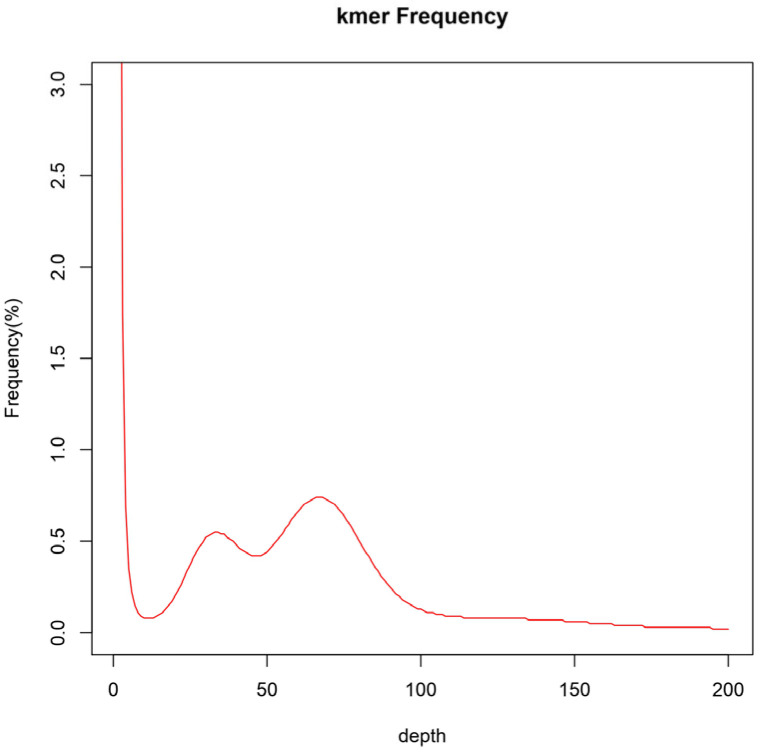
Distribution curve of K-mer (k = 17) of *I. pseudotinctoria*. The *x*-axis is depth and the *y*-axis is the proportion that represents the frequency at that depth divided by the total frequency of all depths.

**Figure 4:**
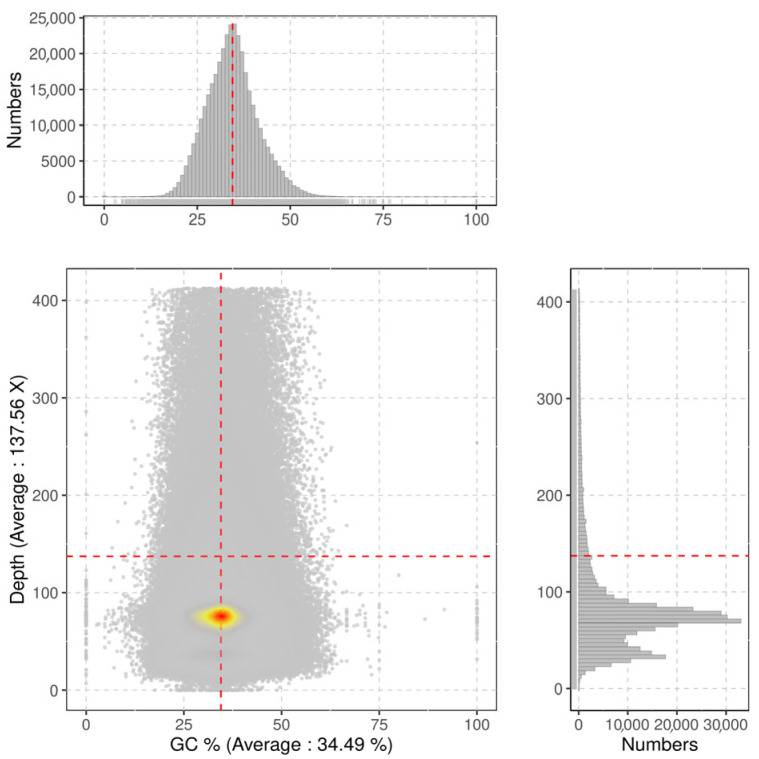
Average sequencing depth and GC content of *I. pseudotinctoria*. The *x*-axis represents the GC content and the *y*-axis is the sequence depth. The distribution of the sequence depth is on the right side, while the distribution of the GC content is at the top. Each dot represents a contig, color from red to yellow and then to gry indicates dot density from high to low.

The complete set of clean reads was employed to predict the genomic attributes of *I. pseudotinctoria* through k-mer analysis. Utilizing the 17-mer frequency distribution, the genome size was appraised at 637 Mb, constituting 69% of the size (920 Mb) estimated via flow cytometry. Moreover, the ratios of heterozygosity and repeat sequence content were determined as 0.98% and 66.30%, respectively (Figure 3 and Table 5). Consequently, it is evident that the genome of *I. pseudotinctoria* aligns with a complex nature, underscored by heightened heterozygosity and repeat sequence. Additionally, the genomic GC content registered at 34.3% (Figure 4).

### 3.4. Identifcation and Verification of SSRs

**Figure 5:**
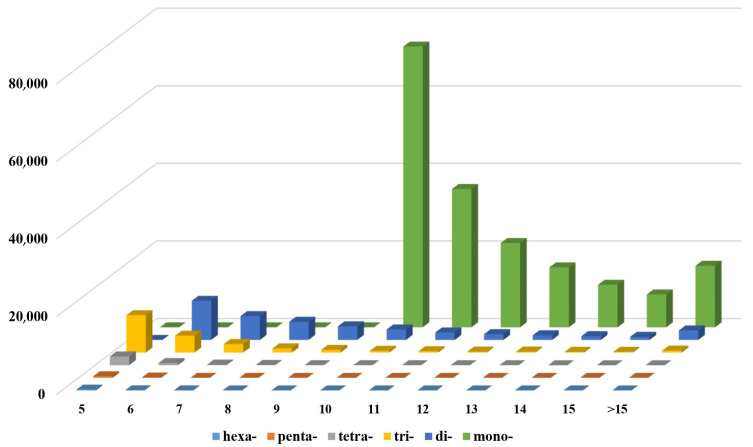
The distribution and frequency of SSR motif repeat numbers. *x*-axis is the number of repeats, *y*-axis is microsatellite class/type according to motif length and z axis is frequency of given microsatellite type.

**Figure 6:**
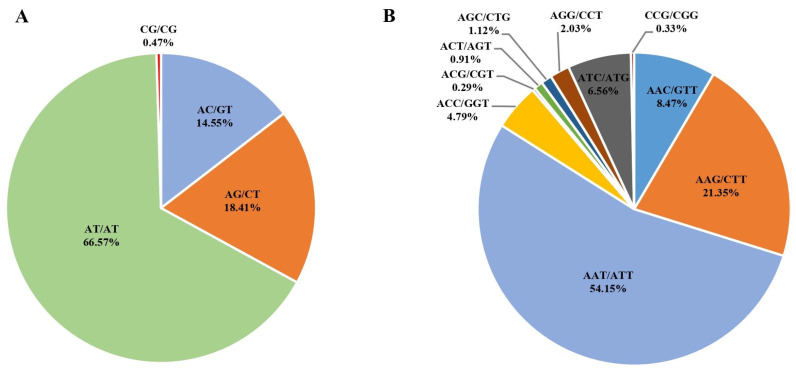
Identification and characteristics of simple repeat sequence motif. (**A**). Percentage of different motifs in dinucleotide repeats in *I. pseudotinctoria*; (**B**). Percentage of different motifs in trinucleotide repeats in *I. pseudotinctoria*.

A total of 240,659 SSRs was discerned within the draft genome sequence of the *I. pseudotinctoria* (Table 6). Notably, mononucleotide SSRs emerged as the most prevalent, constituting 74.50% (180,491) of the total SSR. This was followed by dinucleotide SSRs (35,978; 14.95%), trinucleotide SSRs (20,213; 8.40%), tetranucleotide SSRs (3140; 1.30%), pentanucleotide SSRs (515; 0.21%), and hexanucleotide SSRs (322; 0.13%) (Table 6 and Figure 5). Among the mononucleotide repeats, the AT/AT motif predominated, accounting for 66.57% of the total repeat units. In the dinucleotide context, the most frequent motif observed was AG/CT (18.41%), followed by AC/GT (14.55%) (Figure 6A). For trinucleotide repeats, the prevailing motifs were AAT/ATT, AAG/CTT, AAC/GTT, and AGG/CCT, encompassing proportions of 54.15%, 21.35%, 8.47%, and 2.03%, respectively (Figure 6B).

**Figure 7:**
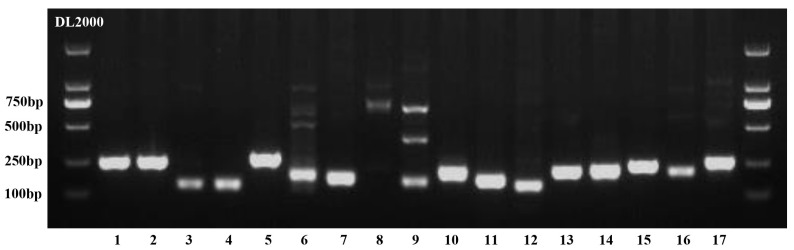
Genomic SSR-PCR amplification products from 17 primer pairs resolved by 2% agarose gel electrophoresis at 120 V.

Given the numerous SSRs identified, we randomly selected 17 tri- and tetranucleotide microsatellite loci from the draft genome of *I. pseudotinctoria* and designed corresponding primer pairs. These selected loci predominantly contained five or six motif repeats. The amplification results demonstrated that 16 out of the 17 SSR loci could be successfully amplified (Figure 7), confirming the reliability and reproducibility of our genomic SSR marker identification.

### 3.5. Gene Prediction and Annotation

**Figure 8:**
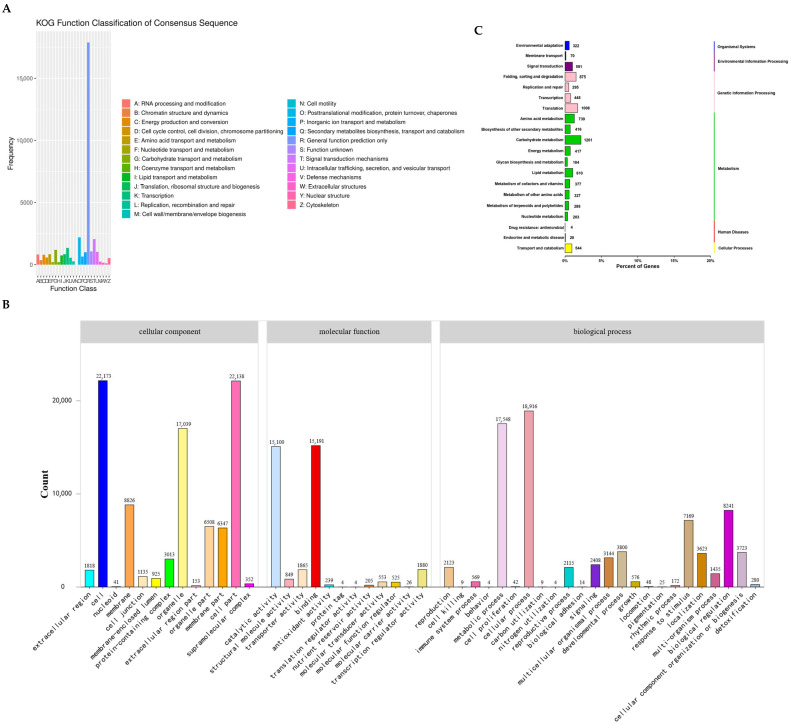
The genes were aligned by BLAST to the KOG, GO, and KEGG database. (**A**) KOG functional categories; (**B**) Gene Ontology classification, genes were assigned to three categories—cellular components, molecular functions, and biological processes; (**C**) gene assignment to KEGG functional categories in *I. pseudotinctoria*.

A comprehensive total of 58,790 unigenes, constituting 98.91% of all unigenes, were meticulously matched and subsequently annotated within the Nr homologous database (Figure S1 and Table S1). Overall, 33,218 putative genes were classified into KOG functional categories (Figure 8A), among which the largest cluster was general function prediction (17,453; 52.54%), followed by post-translational modification (1867; 5.62%), signal transduction mechanisms (1497; 8.58%), and transcription (1086; 6.22%) (Table S2). Utilizing sequence homology, the 28,111 assembled transcripts were systematically assigned into GO terms, including biological process, cellular component, and molecular function (Figure 8B). Within biological processes, the category predominantly represented was cellular processes (18,916; 23.98%), succeeded by metabolic processes (17,548; 20.98%) and biological regulation (8241; 17.59%). For cellular components, the most prevalent category was the cell part (22,173; 24.52%), followed by organelle (17,039; 18.84%) and membrane (8826; 9.76%). In terms of molecular function, two major categories stood out: binding (15,191; 19.98%) and catalytic activity (15,100; 19.87%).

Furthermore, the assignment of putative genes to 131 KEGG pathways yielded a total of 57,636 associations (Figure 8C), among which 10,893 genes were correlated with 20 metabolic pathways. Notably, carbohydrate metabolism featured prominently (1261; 11.58%), closely pursued by translation (3025; 27.8%), amino acid metabolism (739; 6.8%), and lipid metabolism (610; 5.6%). Additionally, organismal systems pathways, environmental information processing, genetic information processing, and metabolism KEGG pathways were linked to 332, 661, 2623, and 4549 genes, respectively.

## 4. Discussion

This study represents a significant advancement in understanding the complex genomics of *I. pseudotinctoria*, a traditional Chinese botanical species that holds paramount economic values due to its applications in forage production and medicinal practices. The relevance of our investigation is underscored by the paucity of genomic information pertaining to this species. This study used flow cytometry to determine the genome size of *I. pseudotinctoria*, yielding a result of 920 Mb (Table 1), whereas K-mer-based survey analysis estimated it to be 637 Mb (Table 5). The genome size measured by flow cytometry is larger than that obtained through K-mer analysis, a trend consistent with findings in other plant species such as *Parrotia* [33], *Sophora alopecuroides* [34], *Cucumis sativus* [35], and *Panax ginseng* [36]. Studies have shown that flow cytometry employs dissociated and stained cell suspensions as test samples, and thus the results can be influenced by plant cellular structures and secondary metabolites [37]. In contrast, K-mer-based survey methods are not affected by endogenous cellular substances; however, during second-generation sequencing, some genomic fragments may be lost due to the fragmentation and assembly processes, resulting in an underestimation of the genome size. Notably, the observed heterozygosity rate of 0.98% and a repeat rate of 66.3% collectively indicated a genome with a high level of heterozygosity and repeat sequence (Figure 3 and Table 5). The resulting genetic diversity, which aligns with the species’ agricultural importance, is poised to enhance its biological adaptability. This propensity for heterogeneity is expected to contribute to the species’ overall genetic diversity, thereby enhancing its capacity to adapt and interact within its environment, which notably corresponds to its significant agricultural role.

Simple sequence repeat (SSR) markers are valuable tools for assessing population structure and genetic diversity within native populations of *I. pseudotinctoria*, as well as for evaluating genetic perturbations caused by non-native conspecifics. A previous study conducted by Fan et al. [38] used amplified fragment length polymorphism (AFLP) technology to investigate the genetic diversity, differentiation, and structure of *I. pseudotinctoria* genotypes. It is important to note that there are significant methodological differences between AFLP (a dominant marker) and SSR (a co-dominant marker) techniques, primarily due to their fundamentally distinct patterns of genetic inheritance [39]. Previous studies have used microsatellite markers to assess genetic disturbance in native populations of *I. pseudotinctoria* resulting from the introduction of non-native conspecifics [40]. However, these studies have limitations in comprehensively evaluating the entire genome of *I. pseudotinctoria*. In the context of this current study, a notable finding is the identification of 240,659 SSR loci within the genome of *I. pseudotinctoria* (Table 6). The observed variations in nucleotide repeat abundance indicated that mono-nucleotide repeats are the most abundant, while pentanucleotide repeats are conspicuously rare (Table 6). These patterns reflect the complex evolutionary forces that have shaped the genomic landscape of this species. Moreover, the prominence of specific sequence motifs, such as AT/AT and AAT/ATT within dinucleotide and trinucleotide repeats, respectively (Figure 6), suggests their potential involvement in critical functional aspects of the genome. Furthermore, SSR markers developed from functional genes are expected to significantly enhance the precision of marker-assisted selection and association mapping [41]. Genome-wide analysis identified 11,027 Pfam-annotated genes harboring SSRs, with enrichment analysis revealing that the top 10 functional domains [e.g., pentatricopeptide *repeats* (*PPRs*), *protein kinases*, and *Myb* genes] were associated with plant growth regulation and stress responses (Figure S2). These findings suggest SSR-containing genes may mediate important biological functions, providing valuable genetic markers for *I. pseudotinctoria* breeding programs.

The impact of GC content on sequencing bias caused by the Illumina sequencing platform was identified as one of the three key factors contributing to this phenomenon [25]. Variations in GC content beyond the optimal range (25–65%) result in uneven se-quencing coverage, thereby compromising the accuracy and completeness of genome assembly [42]. In the present study, the GC content of *I. pseudotinctoria* was found to be moderate, which aligns with the typical range of 30% to 47% that observed in most plant species [1,43]. Consequently, it is postulated that this moderate GC content would not exert a significant influence on the quality of the genome sequencing during the sequencing process [44].

Our investigation goes beyond mere sequence analysis by identifying 58,790 unigenes that align with known genes in widely recognized databases, particularly the GO and KEGG (Figure 8). This expansion enhances our comprehension of the underlying biological mechanisms of *I. pseudotinctoria*, offering insights into its molecular functionalities and potential pathways. The substantial representation of genes within these databases underscores the biological richness of this species, amplifying its significance in both agricultural and medicinal contexts [45]. Notably, our study highlights the presence of numerous genes mapped onto key pathways of KEGG within *I. pseudotinctoria*, potentially shedding light on its ability to synthesize essential amino acids, secondary metabolites, and bioactive compounds. This is particularly relevant when considering the plant’s historical medicinal application. Amino acids, beyond their primary roles, also serve as precursors in the biosynthesis of diverse secondary metabolites [46]. In *I. pseudotinctoria*, secondary metabolites likely play a vital role in protecting the plant against pathogens and herbivores, a trait that is notably consistent with its historical medicinal use [5]. Consequently, the identified genetic repertoire not only provides valuable insights for exploring the realm of secondary metabolites but also holds the potential to unearth distinctive bioactive compounds that might find application in various fields, including phytochemicals with potential health benefits, thus aligning with its medicinal traditions.

## 5. Conclusions

In conclusion, this study not only accomplishes the sequencing of the complete genome of *I. pseudotinctoria* but also lays the groundwork for future studies. The genetic insights garnered herein will serve as a cornerstone for subsequent genome-wide investigations, deepening our comprehension of this species’ biology. Furthermore, the identification of SSR markers holds promise for advancing breeding programs and molecular research. This study substantially contributes to the fields of genomics, botany, and agriculture, ultimately providing a vital resource for the further exploration and utilization of *I. pseudotinctoria*.

## Figures and Tables

**Table 1:** Primer of 17 SSR markers in present study.

Locus	Repeat Motif	Primer Sequence (5′-3′)	
		Forward	Reverse
IP380501	(GTT)5	AATTTTTCCACGGGGTCTTC	GTTGGTTTTATCCGTCGCTT
IP553716	(ATA)5	ATTGGTTGTGTGGACCGAAT	TCAAATTATTCCCTTATTCAAATTCA
IP885913	(ATTT)5	AATACAGGTGAGCAGTGCGA	TGAAATTCCACCACAATGGA
IP1040384	(AAAT)5	AATTGTCCTCGTGTTGTGAGG	AATGGTGCGAATTTTATGCTT
IP1047571	(GAT)5	TCCTAAGCCACCACAAATCC	CCATCTCCTACCTTCCAACTTC
IP1227591	(AGA)5	ACGAATCAGAAGAACAGGGC	TCTCTCACAAACACCGACCA
IP1434377	(GGC)5	TTCGATTTGGATTTGCACTG	AGAATGTTCTGCACCGTTCC
IP2408442	(TATT)7	CGCTGTTTAGGTTAACATTCCA	ACATCCCCATTAACTCAACATAG
IP10099562	(TAC)5	GGCCCTTTTCATTCCTTTTC	ACAACAAGGAGCTCTTCCCA
IP10099615	(CTT)8	TGCAGCAATGATGACATCTG	TTGGCACCACATCAAACAGT
IP10125461	(AAT)7	GGAAGCTACTCTGCATCGGA	CATGCTCATCTCAGGCATGT
IP10125944	(TCT)6	ACCATTAGGCAGAGAGGCAA	TTGCACATGATTCGTTCTCC
IP10130648	(TAC)5	TGTCAGCTTTTGAAGCATGG	GGCCAAAAGTGCAACATTCT
IP10130698	(CCT)6	CCTCCACCTCCCATGTAGAA	AGCCACAAGCTACCTCAGGA
IP10130710	(CAA)5	GGGGTTATTCAGTCCCGTTT	GACGCGACCCAATTGTAACT
IP10130769	(GAT)6	CGAGAGGTTAGGGGGAGATT	CCCACAAATTAAGGGCATGA
IP10130792	(ACA)7	TTGCCACAAATACGCAAAAA	TTCTCAGGTCTGCTCTCGCT

**Table 2:** Statistics of flow cytometry data.

Genome Size (Mb) Mean ± SD	CV (%) of Standard	CV (%) of Sample
920 ± 2	4.73	3.56

**Table 3:** Statistics of sequencing data and quality assessment of *I. pseudotinctoria*.

Number of Raw Reads	Raw Base (Gbp)	Clean Base (Gbp)	Q20 (%)	Q30 (%)	GC Content (%)
365,359,150	54.804	48.952	98.18	93.51	35.96

Abbreviations: Q20 percentage of bases with quality value ≥ 20, Q30 percentage of bases with quality value ≥ 30.

**Table 4:** Statistics of assembled genome sequences for *I. pseudotinctoria*.

Total Length (bp)	Total Number	Max Length (bp)	N50 Length (bp)	N75 Length (bp)	GC Content (%)
431,452,197	553,021	90,236	3506	763	34.3

**Table 5:** Statistical data from the 17-mer analysis.

Kmer	Depth	n_kmer	Genome Size	Heterozygous Ratio	Repeat Sequence Content
17	68	43,729,058,443	6.37 × 10^8^	0.98%	66.30%

**Table 6:** Simple sequence repeat types detected in the *I. pseudotinctoria*.

Searching Item	Number	Ratio (%)
Total number of sequences examined	548,189	
The total size of examined sequences (bp)	430,490,629	
Total number of identifed SSRs	240,659	100
Number of SSR containing sequences	122,195	50.78
Number of sequences containing more than 1 SSR	46,239	19.21
Number of SSRs present in the compound formation	28,086	11.67
Mononucleotide	180,491	74.50
Dinucleotide	35,978	14.95
Trinucleotide	20,213	8.40
Tetranucleotide	3140	1.30
Pentanucleotide	515	0.21
Hexanucleotide	322	0.13

## Data Availability

Raw data and the genome assembly from this study were deposited in China National GeneBank Database under the ID: CNP0007515.

## Supplementary Materials

The following supporting information can be downloaded at: https://www.mdpi.com/article/10.3390/genes16090991/s1, Figure S1: Distribution of *I. pseudotinctoria* gene in Nr homologous database; Figure S2: Top 10 functional terms of genes containing SSR; Table S1: Basic function annotation; Table S2: KOG functional categories.
