# Supplementary figures and images for: Genome Survey Sequencing of Indigofera pseudotinctoria and Identification of Its SSR Markers
Source: Genes (Basel). 2025 Aug 23;16(9):991. doi: 10.3390/genes16090991 (PMC12469494; doi:10.3390/genes16090991)

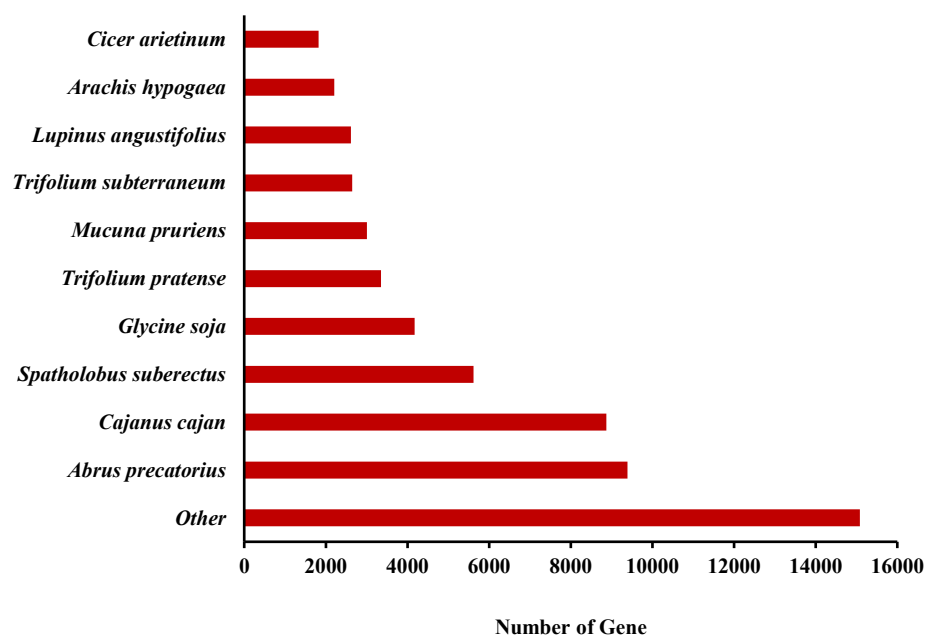

**Figure S1.** Distribution of *I. pseudotinctoria* gene in Nr homologous database

Supplement: Supplementary file 1 [file genes-16-00991-s001.zip › Figure S1. Distribution of I. pseudotinctoria gene in Nr homologous database.pdf]

Top 10 Pfam terms in SSR-containing genes

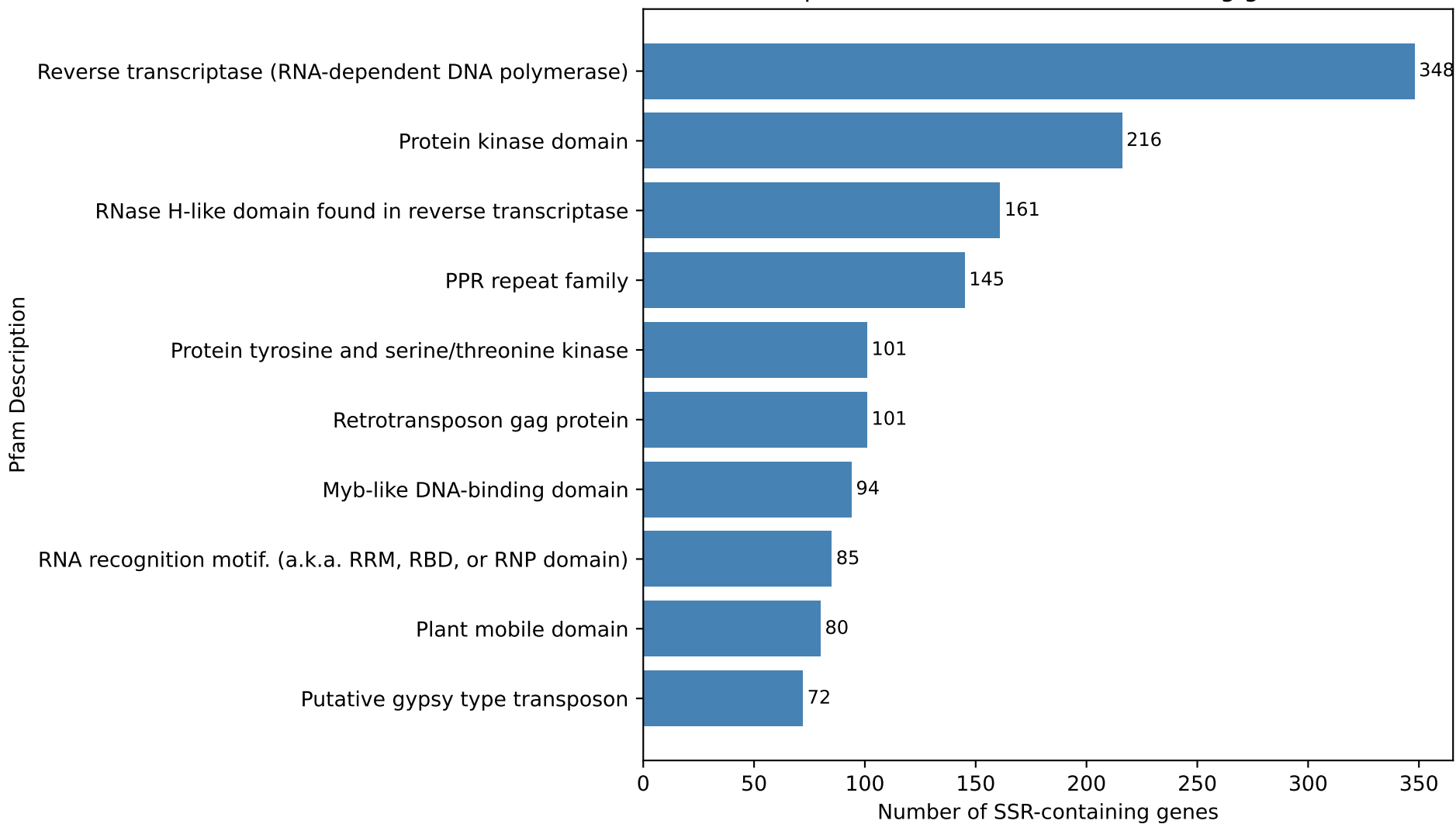

Supplement: Supplementary file 1 [file genes-16-00991-s001.zip › Figure S2. Top 10 functional terms of genes containing SSR.pdf]
